# Long-term, up to 18 months, protective effects of the angiotensin II receptor blocker telmisartan on Epirubin-induced inflammation and oxidative stress assessed by serial strain rate

**DOI:** 10.1186/2193-1801-2-198

**Published:** 2013-04-30

**Authors:** Mariele Dessì, Clelia Madeddu, Alessandra Piras, Christian Cadeddu, Giorgia Antoni, Giuseppe Mercuro, Giovanni Mantovani

**Affiliations:** Department of Medical Sciences: Medical Oncology, University of Cagliari, Cagliari, Italy; Department of Medical Sciences: Cardiovascular Diseases, University of Cagliari, Cagliari, Italy; Policlinico Universitario, SS 554, Km 4500, 09042 Monserrato, Italy

**Keywords:** Epirubicin-induced cardiotoxicity, Cytokines, Oxidative stress, RAS, Telmisartan

## Abstract

**Purpose:**

The primary objective of the present study was to show the long lasting cardioprotective activity, at different time-points, up to 18 month-follow-up, of telmisartan in preserving the systolic function (assessed as Strain Rate-SR) in cancer patients treated with EPI both in the adjuvant and metastatic setting; the secondary objective was to confirm the correlation of the cardioprotective activity of telmisartan with a reduction of inflammation and oxidative stress induced by EPI.

**Methods:**

Phase II single blind placebo-controlled randomized trial. Sample size 50 patients per arm: based on a pre-planned interim analysis for early stopping rules, the study was discontinued for ethical reasons at 49 patients. Cardiovascular disease-free patients with cancer at different sites eligible for EPI-based treatment randomized to: telmisartan n = 25 or placebo n = 24. Echocardiography Tissue Doppler imaging (TDI) strain and strain rate was performed, serum levels of proinflammatory cytokines (IL-6, TNF-α) and oxidative stress (reactive oxygen species, ROS) were assessed at baseline, every 100 mg/m^2^ EPI dose and at 6-, 12- and 18-month follow-up (FU).

**Results:**

Significant SR peak reduction in both arms was observed at t_2_ (cumulative dose EPI 200 mg/m^2^) *vs* t_0_. Conversely, at t_3_, t_4_, 6-, 12- and 18-month FU SR increased towards normal range in the telmisartan arm, while in the placebo arm SR remained significantly lower. Differences between SR changes in the placebo and telmisartan arm were significant from t_3_ up to 18 month-FU. IL-6 and ROS increased significantly in the placebo arm at t_2_ but did not change in the telmisartan arm. A significant (p < 0.05) correlation between changes of SR vs IL-6 and ROS was observed.

**Conclusions:**

Our results suggest that the protective effect of telmisartan is long lasting, probably by ensuring a permanent (at least up to 18-month FU) defense against chronic or late-onset types of anthracycline-induced cardiotoxicity.

## Introduction

Anthracyclines (ANT) are among the most effective drugs against cancer and are used in a wide spectrum of malignancies. Regrettably, their clinical use is limited by the occurrence of dose-related cardiotoxicity (Paulides and Wojnowski 2007).

Several studies have shown that anthracycline-induced cardiotoxicity (CTX) is at least partially mediated by chronic inflammation and oxidative stress: indeed, proinflammatory cytokines interleukin-6 (IL-6), tumor necrosis factor-alpha (TNF-α) and Reactive Oxygen Species (Thompson, et al.) all play a central role (Meldrum, et al. 1998, Kupatt, et al. 1999). It has also been shown that the use of a conventional cardioprotective agent, such as dexrazoxane, together with chemotherapy, reduces the expression of the NRF-2 gene (responsible for oxidative stress response), which is over-expressed in patients receiving ANT alone (Thompson, et al. 2010).

A recent and growing mass of evidence shows the involvement of the renin-angiotensin-system (RAS) in the ANT-induced CTX. The angiotensin II plays a crucial role not only as a vasoconstrictor agent but also as a mitogenic factor by interacting with angiotensin II type-1 receptors (AT1Rs) in the cardiovascular myocytes (Toko, et al. 2002). Cardiac dysfunction after doxorubicin was not shown in the knockout rat for the AT1R gene, a finding confirmed by the absence of apoptosis and myofibrillar damage (Soga, et al. 2006). In a recent study, the cardioprotective effect of angiotensin receptor blocker (AT1Rs) telmisartan has been shown in rats exposed to ANT (Iqbal, et al. 2008). The authors argued that the effect was sustained by a decrease of oxidative stress, which in turn is able to reduce the structural damage of cardiomyocytes. As regards the possible role of ARBs in mitogenesis and angiogenesis, it was observed that these drugs were able to suppress the signal transduction mediated by growth factors, such as the epidermal growth factor (EGF), through the AT1R antagonism (Ishiguro, et al. 2007). Furthermore, the ARB telmisartan was shown to be able to inhibit the proliferation of prostate cancer cells through the activation of the peroxisome proliferator-activated receptor-γ (PPAR-γ) (Funao et al. 2008).

We previously identified an EPI-induced early myocardial dysfunction, detected after low dose (200 mg/m^2^) of EPI (Mercuro, et al. 2007). This dysfunction was shown to be correlated to a significant increase of several biological markers of inflammation and oxidative stress and persisted throughout the treatment with EPI and up to 18 month follow-up (Mantovani, et al. 2008).

In a previous phase II placebo-controlled study we used telmisartan in order to prevent EPI-induced myocardial damage (Cadeddu, et al. 2010). We aimed to exploit the ability of this drug to inhibit the production of superoxide radicals by mitochondrial NADPH-dependent oxidase and xanthine oxidase (Wenzel, et al. 2008) and to, at least partially, antagonize the PPAR-γ activation (Stephen, et al. 2004). Our study showed that telmisartan was able to reduce EPI-induced oxidative stress/chronic inflammation and to reverse early myocardial impairment (Cadeddu, et al. 2010).

The primary objective of the present study was to show the long lasting cardioprotective activity, at different time-points, up to 18 month-follow-up, of telmisartan in preserving the systolic function (assessed as Strain Rate-SR) in cancer patients treated with EPI both in the adjuvant and metastatic setting; the secondary objective was to confirm the correlation of the cardioprotective activity of telmisartan with a reduction of inflammation and oxidative stress induced by EPI.

## Patients and methods

### Patient population and study protocol

From September 2008 to October 2009, 49 consecutive eligible patients (male/female ratio: 12/37) with histologically confirmed tumors at different sites, previously untreated were enrolled. All eligible patients were included in the study. The majority of patients (40 patients) had early stage cancer and therefore were treated in the adjuvant setting while the remaining patients (9 patients) had locally advanced or metastatic disease and were treated in the metastatic setting. Patients eligible for EPI-based chemotherapy regimen were randomized and treated with a combination chemotherapy schedule containing EPI. The great majority of patients followed a schedule of 100 ± 30 mg/m^2^ every three weeks, while the remaining patients followed a schedule other than 100 ± 30 mg/m^2^ every three weeks (for example 30 mg/m^2^ weekly). Patients were treated up to a maximal cumulative dose of EPI 400 ± 30 (SD) mg/m^2^, according to the international standardized protocols for EPI-based administration.

Inclusion criteria were the following: patients aged 18–70 yo; blood pressure within the normal range (80/120); echocardiographic LVEF value ≥55%; SR value in the normal range (range: 1.7-2.1 cm/sec); Eastern Cooperative Oncology Group (ECOG) performance status score of 0–2 (Oken, et al. 1982); normal hepatic and renal function (bilirubin ≤ 1.5 mg/dl, creatinine ≤ 2.0 mg/dl); no concomitant medications known to interfere with inflammatory and oxidative stress parameters such as NSAIDs, aspirin, Cox-2 inhibitors.

Patients were not eligible if they had a history of cardiac disease, hypertension, diabetes and/or had been previously treated with mediastinal radiation therapy. The study was one-Institution “independent”, single blind randomized, placebo-controlled trial; it was approved by the Institutional Ethics Committee (“Azienda Ospedaliero Universitaria” of Cagliari, Italy) and written informed consent was obtained by all patients included in the study. The trial was carried out in accordance with Good Clinical Practices and the Helsinki Declaration.

A blind randomization was performed: 25 patients were randomized by a block randomization technique to the telmisartan arm and 24 to the placebo arm. The random allocation was generated by a data manager, patients were enrolled by a physician (oncologist) and were then assigned to intervention by another physician (oncologist). None of the above mentioned people were involved in the data evaluation.

Patients were treated with telmisartan (Micardis, Boehringer-Ingelheim, Milan, Italy) 40 mg, 1 tablet/day, or placebo starting one week before the beginning of EPI treatment and up to six months after EPI discontinuation. The telmisartan dose was chosen according to the safety and efficacy shown in our previously published study (Cadeddu, et al. 2010). The placebo tablets were supplied from the Institutional Pharmacy and were identical looking and tasting to telmisartan.

### Clinical and laboratory assessments

At enrollment, before randomization, as well as after each subsequent administration of EPI, patients underwent physical examination, blood pressure measurement, 12-lead electrocardiogram and echocardiographic analysis (conventional and Tissue Doppler Imaging, TDI, technique). The following laboratory tests were carried out: blood and platelet count, BUN, uric acid, creatinine, blood and urine electrolytes, direct and indirect bilirubin, AST, ALT, γGT, alkaline phosphatase, iron, ferritinemia and blood transferrin. Blood samples were collected for the assessment of circulating levels of proinflammatory cytokines (IL-6 and TNF-α), ROS and antioxidant enzymes glutathione peroxidase (GPx) and superoxide dismutase (SOD) from all patients. The instrumental and laboratory variables were assessed at baseline (t_0_), 7 days after reaching the EPI dose of 100, 200, 300, 400 mg/m^2^ (t_1_, t_2_, t_3_ and t_4_, respectively) and at 6, 12 and 18 month-follow up. The reported doses of EPI are to be always intended as cumulative.

### Conventional echocardiography and TDI

Echocardiographic images were recorded using a commercially available system equipped with TDI, Strain (S) and Strain Rate (Khasraw et al. 2012) imaging (Toshiba APLIO CV ultrasound system-SSA 770A/CV; Toshiba Corp., Tochigi, Japan). LVEF was obtained from the apical 4-and 2-chamber views according to Simpson’s rule and was considered abnormal under 55%.

Conventional echocardiography parameters such as left ventricular end diastolic diameter (LVEDD) and atrial dimensions were assessed in both arms.

We performed a pulsed wave Doppler (PWD) examination of the LV inflow from the 4-chamber view with the sample volume placed between the mitral leaflet tips and the early (E) and late (A) diastolic peak velocities; E deceleration time (DecT) was measured and then the E/A ratio was derived. We evaluated the longitudinal function using pulsed TDI at the mitral annulus, placing the sample volume in the basal segment of the interventricular septum (IVS) from the apical 4-chamber view: peak velocities in systole (Jones et al. 2009), isovolumic relaxation time (IVRT), early (Em) and late (Am) diastole were measured. LV longitudinal function was evaluated from raw data; myocardial S and SR were also quantified in the IVS. The same experienced echocardiographer carried out all examinations of each individual patient. To reduce inter-observer variability, all echocardiographic data were randomly read by a second experienced observer and an average value for each measurement was calculated. Reproducibility of TDI parameters in our laboratory had been previously documented (Cadeddu, et al. 2010).

### Inflammatory and oxidative stress markers

In all patients, a blood sample was obtained by venipuncture of antecubital vein at 8 a.m., after overnight fasting. Levels of IL-6 and TNF-α were determined by enzyme-linked immunosorbent assay (Immunotech, Marseille, France) and expressed in pg/ml. ROS blood levels were determined on fresh heparinized blood samples using the free oxygen radicals test (FORT). Results are expressed as FORT units (U), where 1 FORT U corresponds to 0.26 mg/l of H_2_O_2_. The erythrocyte antioxidant enzymes GPx and SOD were measured by photometer using a commercially available kit (Ransod, Randox Lab, Crumlin, United Kingdom) and expressed as U/l and U/ml, respectively.

### Timing of echocardiographic and biomarker assessments

Echocardiographic and biomarker assessments were carried out according to the schedule reported in Table 1.

**Table 1:** **Timing of echocardiographic and biomarker assessments**

t_0_	t_1_	t_2_	t_3_	t_4_
Before EPI	week 1	week 4	week 7	week 10
For EPI dose other than 100 ± 30 mg/m2 every three weeks				
**t**_**0**_	**t**_**1**_	**t**_**2**_	**t**_**3**_	**t**_**4**_
Before EPI	1 week after reaching 100 ± 30 mg/m^2^	1 week after reaching 200 ± 30 mg/m^2^	1 week after reaching 300 ± 30 mg/m^2^	1 week after reaching 400 ± 30 mg/m^2^

For EPI dose 100 ± 30 mg/m_2_ every three weeks.

### Statistical analysis

Considering an α type error of 0.05, a β type error of 0.10 and a difference of SR changes between arms of 10% of the primary endpoint (SR change) as clinically meaningful, 50 patients should have been enrolled in each arm.

An interim analysis on the basis of the early-stopping rules was pre-planned. The futility stopping rule was defined for a two-sided p value testing the superiority of telmisartan in terms of SR change. A p value < 0.05 would favor telmisartan, whereas a p value > 0.05 would favor placebo. The p value for stopping the study for efficacy was 0.01. At the time of stopping the study we had reached a p value < 0.05.

Treatment arms were compared by the Student’s *t*-test for changes. Differences between values measured at different times (different EPI doses) and at 6-, 12-and 18-month FU were calculated by the ANOVA test, with Bonferroni correction.

The correlation between instrumental (TDI) and laboratory variables was assessed by Pearson’s *t*-test (or Spearman’s *t*-test for non-parametric variables).

Significant relationships were then examined by multivariate linear regression analysis.

Results were considered significant for p values ≤0.05. Data are reported as mean ± SD. Statistical analysis was performed using SPSS version 14 for Windows.

## Results

**Figure 1:**
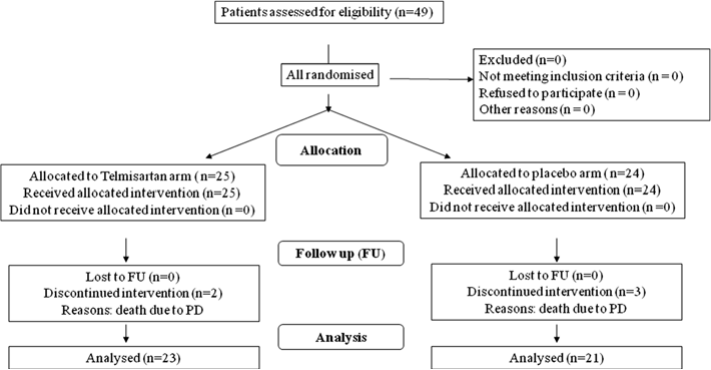
**Consort Diagram.**

Clinical characteristics of patients in each arm were well balanced and are summarized in Table 2. All patients reached the scheduled cumulative EPI dose of 400 mg/m^2^. As regards tumor history, it should be noted that overall 5 patients died at 5 ± 2 months after the end of EPI treatment due to disease progression (PD): 2 patients in the telmisartan arm, 3 patients in the placebo arm. The Consort Diagram is reported in Figure 1. Moreover, at 18 month-FU PD occurred in 1/23 patients in the telmisartan arm and in 2/21 patients in the placebo arm.

**Table 2:** **Patient clinical characteristics**

	TEL	PLA
Patients evaluated	25	24
Male/female	6/19	6/18
Age, years: mean ± SD (range) 59 ± 14 (27–78)	52.9 ± 9	53 ± 10
Alive	23	21
Dead	2	3
**Tumor type**	**TEL**	**PLA**
Breast	8	10
Endometrium	12	9
Non-Hodgkin’s lymphoma	1	2
NSCLC	0	1
Ovary	4	1
Salivary gland	0	1
**Stage**	**TEL**	**PLA**
I	14	13
II	6	7
III	4	3
IV	1	1
**ECOG/PS**	**TEL**	**PLA**
0	18	15
1	6	4
2	1	5

*Abbreviations. ECOG PS: Eastern Cooperative Oncology Group performance status; NSCLC: non-small cell lung cancer.*

### Serial assessment of 12-lead electrocardiograms over time

At ECG monitoring, a normal morphology was observed throughout the treatment in 33 patients; in 16 patients (9 in the telmisartan and 7 in the placebo arm) we observed widespread and unspecified changes during the ventricular repolarization phase concurrent with t_2_ with no significant differences between the telmisartan and placebo arm.

### Conventional echocardiography and TDI

In the placebo arm, at t_3_ and t_4_ we observed a significant LV diastolic impairment, represented by a reduction in the E/A ratio at PWD (p < 0.05). In the telmisartan arm we observed a slight reduction in the E/A ratio, which, however, did not reach statistical significance. No significant abnormalities of LVEF and DecT were found in any of the two arms throughout the treatment (Table 3).

**Table 3:** **Conventional ecocardiographic parameters of systolic and diastolic function in both arms**

Conventional echo	t_0_ (n = 49)	t_2_ (n = 49)	t_3_ (n = 49)	t_4_ (n = 49)	12-Month FU (n = 44)	18-Month FU (n = 44)
LVEF						
PLA	66 ± 5%	68 ± 6%	66 ± 5%	66 ± 5%	67 ± 5%	65 ± 7%
TEL	66 ± 7%	67 ± 6%	68 ± 4%	70 ± 6%	68 ± 4%	66 ± 6%
DecT						
PLA	0.22 ± 0.04	0.24 ± 0.05	0.22 ± 0.02	0.23 ± 0.04	0.22 ± 0.03	0.23 ± 0.04
TEL	0.19 ± 0.04	0.21 ± 0.04	0.20 ± 0.02	0.21 ± 0.03	0.21 ± 0.04	0.20 ± 0.05
E/A						
PLA	1.13 ± 0.14	1.08 ± 0.12	0.92 ± 0.05*	0.90 ± 0.06*	1.06 ± 0.42	1.06 ± 0.29
TEL	0.96 ± 0.12	0.86 ± 0.08	0.83 ± 0.07	0.95 ± 0.14	0.87 ± 0.31	0.89 ± 0.24

*LVEF*, left ventricle ejection fraction; *DecT*, deceleration time; *E/A*, early and late diastolic peak velocity ratio; t_0_, baseline; t_2_, 200 mg/m2 EPI; t_3_, 300 mg/m2 EPI; t_4_, 400 mg/m2 EPI; * p < 0.05 vs t_0_.

Conventional echocardiography parameters, i.e. left ventricular end diastolic diameter (LVEDD) and atrial dimensions were in the normal range and not different between arms at baseline and did not change during treatment up to 18 month-FU (data not shown).

TDI echocardiographic analysis showed in the placebo arm a LV diastolic impairment, highlighted by a reduction in the Em/Am ratio measured in the basal portion of IVS, first recognized at t_2_ (p‹0.05; Table 4): this worsened function persisted throughout the treatment, at t_3_ (p‹0.05) and t_4_ (p‹0.05; Table 4), whereas in the telmisartan arm the diastolic impairment did not occur. At 12 and 18 month-FU Em/Am ratio returned within the t_0_ range in the placebo arm. The other TDI parameters (Em, Sm, S) did not show any significant changes during treatment up to 18 month-FU in any of the two arms (Table 4).

**Table 4:** **TDI ecocardiographic parameters of systolic and diastolic function in both arms**

TDI echo	t_0_ (n = 49)	t_2_ (n = 49)	t_3_ (n = 49)	t_4_ (n = 49)	12-Month FU (n = 44)	18-Month FU (n = 44)
E_m_						
PLA	8.66 ± 4.90	8.64 ± 6.07	7.73 ± 4.90	7.54 ± 3.50	7.88 ± 2.07	7.65 ± 1.66
TEL	7.89 ± 2.14	7.33 ± 2.45	7.53 ± 2.17	6.93 ± 1.46	7.74 ± 1.58	7.49 ± 1.23
E_m_/A_m_						
PLA	1.13 ± 0.26	0.85 ± 0.35*	0.72 ± 0.30*	0.75 ± 0.32*	0.96 ± 0.27	0.87 ± 0.33
TEL	0.90 ± 0.11	0.84 ± 0.07	0.85 ± 0.05	0.71 ± 0.23	0.78 ± 0.28	0.75 ± 0.27
S_m_						
PLA	7.15 ± 0.65	7.28 ± 1.71	7.28 ± 0.82	6.83 ± 0.69	7.25 ± 1.37	6.82 ± 0.87
TEL	7.33 ± 1.76	7.09 ± 1.21	7.04 ± 1.12	7.29 ± 1.08	7.33 ± 1.22	6.61 ± 0.93
Strain (S)						
PLA	20.89 ± 1.96	20.75 ± 2.06	18.00 ± 2.55	18.65 ± 1.25	16.78 ± 2.22	17.78 ± 2.73
TEL	22.80 ± 1.54	21.20 ± 1.86	20.40 ± 0.94	19.90 ± 0.92	19.64 ± 1.82	19.18 ± 1.70
TDI echo	t_0_ (n = 49)	t_2_ (n = 49)	t_3_ (n = 49)	t_4_ (n = 49)	12-Month FU (n = 44)	18-Month FU (n = 44)
E_m_						
PLA	8.66 ± 4.90	8.64 ± 6.07	7.73 ± 4.90	7.54 ± 3.50	7.88 ± 2.07	7.65 ± 1.66
TEL	7.89 ± 2.14	7.33 ± 2.45	7.53 ± 2.17	6.93 ± 1.46	7.74 ± 1.58	7.49 ± 1.23
E_m_/A_m_						
PLA	1.13 ± 0.26	0.85 ± 0.35*	0.72 ± 0.30*	0.75 ± 0.32*	0.96 ± 0.27	0.87 ± 0.33
TEL	0.90 ± 0.11	0.84 ± 0.07	0.85 ± 0.05	0.71 ± 0.23	0.78 ± 0.28	0.75 ± 0.27
S_m_						
PLA	7.15 ± 0.65	7.28 ± 1.71	7.28 ± 0.82	6.83 ± 0.69	7.25 ± 1.37	6.82 ± 0.87
TEL	7.33 ± 1.76	7.09 ± 1.21	7.04 ± 1.12	7.29 ± 1.08	7.33 ± 1.22	6.61 ± 0.93
Strain (S)						
PLA	20.89 ± 1.96	20.75 ± 2.06	18.00 ± 2.55	18.65 ± 1.25	16.78 ± 2.22	17.78 ± 2.73
TEL	22.80 ± 1.54	21.20 ± 1.86	20.40 ± 0.94	19.90 ± 0.92	19.64 ± 1.82	19.18 ± 1.70

*E*_*m*_,TD early diastolic peak velocity; *E*_*m*_*/A*_*m*_,TD early and late diastolic peak velocity ratio; *S*_*m*_,TD systolic peak velocity; * p < 0.05 vs t_0_.

**Figure 2:**
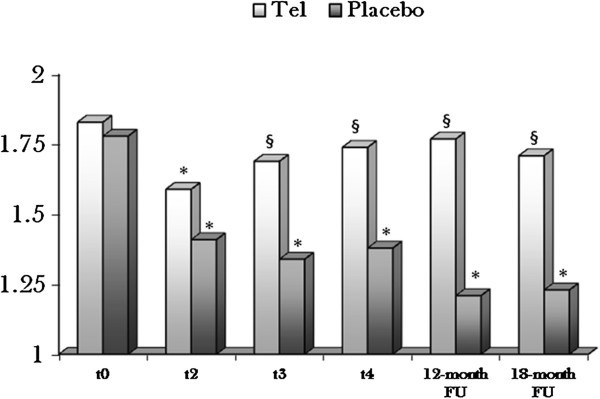
**Strain Rate analysis with TDI in the two arms.**

A significant reduction of the SR peak both in the telmisartan and placebo arm was observed at t_2_ (cumulative dose of 200 mg/m^2^ of EPI) in comparison to t_0_ (1.45 ± 0.33 s-1 vs 1.54 ± 0.35 s-1; NS). Conversely, at t_3_ (300 mg/m^2^ EPI), t_4_ (400 mg/m^2^ EPI) and onwards the SR increased reaching the normal range only in the telmisartan arm, whilst in the placebo arm the SR remained significantly lower as compared to t_0_ (baseline). The differences between SR changes in the placebo and telmisartan arm were significant at t_3_, t_4_ and at 6,12 and 18 month-FU (Figure 2).

### Inflammation and oxidative stress markers

**Figure 3:**
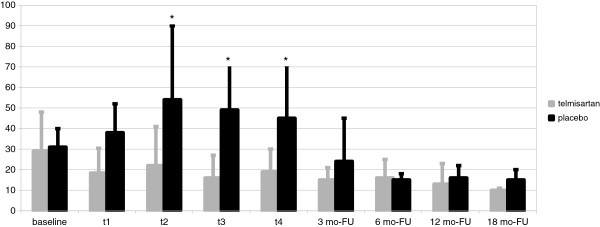
**Serum levels of IL-6 (pg/ml) during the EPI treatment and FU in the telmisartan and placebo arms.**

Serum levels of IL-6 increased significantly in the placebo arm at t_2,_ t_3_ and t_4_, in comparison to baseline (p < 0.05), but remained unchanged in the telmisartan arm (p = 0.356). Thus, a significant difference was observed from t_2_ to t_4_ between the two arms. No difference was found between the two arms from 3 month-FU onward (Figure 3).

**Figure 4:**
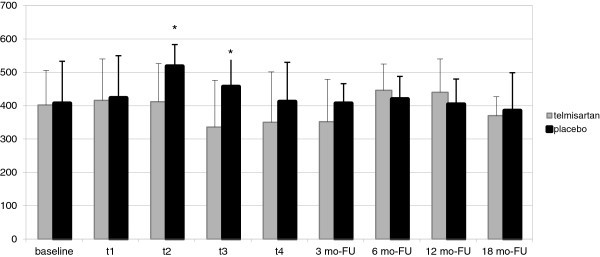
**Blood levels of ROS (FORT U) during the EPI treatment and FU in the telmisartan and placebo arms.**

Blood levels of ROS increased significantly in the placebo arm at t_2_ and t_3_ in comparison to baseline (p = 0.016), whilst remained unchanged in the telmisartan arm (p = 0.319) Thus, a significant difference was observed at t_2_ and t_3_ between the two arms (Figure 4).

The remaining laboratory parameters (TNFα, GPx and SOD) did not show significant changes in either of the two arms throughout the study (Table 5).

**Table 5:** **Inflammation and oxidative stress markers in both arms**

Parameters	t_0_ (n = 49)	t_2_ (n = 49)	t_3_ (n = 49)	t_4_ (n = 49)	12-Month FU (n = 44)	18-Month FU (n = 44)	P - value (ANOVA test)
TNFα							
PLA	30.1 ± 9.0	47.1 ± 4.6	30.0 ± 14.4	25.8 ± 8.6	30.0 ± 14.4	8.3 ± 8.4	0.32
TEL	23.5 ± 5.5	22.3 ± 10.6	35.1 ± 8.6	25.9 ± 4.2	35.1 ± 8.6	12.1 ± 2.9	0.56
GPx							
PLA	7.386 ± 3.041	6.898 ± 1.552	9.427 ± 3.078	10.232 ± 1.875	9.427 ± 3.078	11669 ± 3336	0.56
TEL	7.415 ± 2.182	6.381 ± 2.137	7.263 ± 2.998	7.479 ± 1.769	7.263 ± 2.998	8507 ± 2316	0.88
SOD							
PLA	130.0 ± 9.2	138.0 ± 2.1	193.0 ± 41.0	148.0 ± 38.0	135.0 ± 26.0	134 ± 31	0.32
TEL	126.1 ± 41.6	150.0 ± 37.0	140.0 ± 40.0	145.0 ± 74.0	150.0 ± 37.0	169.5 ± 70	0.95

*TNF*, tumor necrosis factor; *GPx*, glutathione peroxidase; *SOD*, superoxide dismutase.

### Correlations between echocardiography and biomarkers

Both in the placebo and in the telmisartan arm we correlated the changes of ΔSR (calculated as Δ by subtracting the values at t_2_ from values at baseline) to changes in plasma levels of inflammation and oxidative stress markers. We found a significant correlation between ΔSR, increase in IL-6 (r = 0.58, p = 0.001) and in ROS (r = 0.51, p = 0.032) only in placebo arm.

### Safety

Telmisartan was well tolerated by all patients throughout the study: however, a significant hypotension episode (blood pressure values < 95/50 mmHg) was observed only once in 2 patients (approximately corresponding to t_2_ and t_3_). For this event, the telmisartan dose was reduced from 40 mg to 20 mg/day for 2 subsequent weeks and thereafter the full dose of 40 mg/day was re-established.

As for EPI-related side effects, the only significant adverse event was a grade 3/4 neutropenia observed at t_3_ in 6 patients, who needed the administration of Granulocyte Colony-Stimulating Factor and a postponement of the subsequent EPI-based cycle.

## Discussion

The present study confirms the results reported in our previous work (Dessi, et al. 2011) and moreover adds important novel findings: i) a reduction in the SR peak at t_2_ in both treatment arms with no statistical difference between the two arms; ii) a persistent reduction in the SR peak in the placebo arm at t_3_ and t_4_ and, more importantly, a reduction of SR peak persisting also at 18-month FU; iii) a recovery of the SR peak in the telmisartan arm which, starting from t_3_, reaches values within the range of t_0_ which are persistent up to the 18-month FU (data shown for the first time in the present study); iv) a significant increase of serum levels of IL-6 in the placebo arm from t_0_ to t_2_, and a subsequent decrease to the baseline range up to 18-month FU (the latter is a new finding), whereas serum levels of IL-6 in the telmisartan arm remained unchanged from t_0_ to 18-month FU; v) blood levels of ROS show a super imposable pattern to that of IL-6; and vi) changes in the SR peak, an echocardiographic equivalent of early myocardial systolic dysfunction revealed by TDI, correlate with changes in the levels of IL-6 and ROS, which are indicative of the body inflammatory and oxidative stress status, in both arms. Based on the pre-planned early stopping rules (see Statistical Analysis section) the study was discontinued after 49 patients had been enrolled in the study, due to ethical reasons, for the evident superiority of the telmisartan arm.

The findings of the present study confirms that AT1R blockade by telmisartan, administered 1 week before and throughout the duration of EPI treatment, is able to initially (t_2_) reduce and later (t_3_ and t_4_) reverse EPI-induced cardiac abnormalities. This effect is long-lasting and persisted at 18 month FU. Moreover, telmisartan co-administration also prevents increases in IL-6 and ROS levels after EPI administration.

In previous reports, we found that a measurable decline in the SR peak, currently regarded as the earliest sign of subclinical CTX, may be detected in EPI-treated patients long before the clinical evidence of heart failure (Mercuro, et al. 2007). The subtle systolic impairment appeared after 200 mg/m^2^ EPI, a dose which was, until recently, considered insufficient to induce cardiac injury (Jensen, et al. 2002). Moreover, a progressive EPI-induced myocardial dysfunction, which was present even at the 18-month FU in a population of patients not treated with cardioprotective drugs (Mantovani, et al. 2008), was not noted in the present study in the telmisartan arm. As reported above, telmisartan was shown to reverse the early myocardial dysfunction observed at 200 mg/m^2^ EPI and, importantly, its beneficial cardioprotective effect persisted up to the 18-month FU, i.e., 18 months after discontinuation of EPI chemotherapy and 12 months after the end of telmisartan coverage.

A large body of evidence has confirmed the role of the AT1Rs in mediating the damage caused by myocardial ischemia/reperfusion, resulting from acute ANT-induced CTX (Jalowy, et al. 1998, Ferreira, et al. 2008). Accordingly, ANTs were found to induce myofibrillar loss, increase the number of apoptotic cells and significantly impair cardiac function in control mice, but not in AT1R-knockout mice or in animals treated with an AT1R antagonist (Toko, et al. 2002). This evidence suggests that an ARB, such as telmisartan, may be able to prevent, at dosages over 200 mg/m^2^ EPI, ANT-induced CTX.

In a recent study, a protective effect of telmisartan against acute ANT-induced CTX was shown in rats: pre-treatment with the ARB telmisartan elicited a normalization of significant biochemical parameters and reduced cardiac tissue damage (Iqbal, et al. 2008).

Therefore, the present study supports the previously reported role of the RAS in the pathophysiology of chemotherapy-induced CTX; in particular, it also demonstrates for the first time, in a clinical trial, the anti-inflammatory and antioxidant properties of telmisartan, previously observed only in pre-clinical models (Cianchetti, et al. 2008). Moreover, the beneficial effect shown by telmisartan may be explained by its multiple therapeutic characteristics. Indeed, telmisartan is a unique ARB with selective PPAR-γ-modulating activity which affects nitric oxide bioavailability thus leading to its anti-inflammatory, antioxidant and anti-proliferative effects on vascular wall cells (Yamagishi and Takeuchi 2005). Telmisartan was also shown to be able to increase the number of regenerative endothelial progenitor cells and improve endothelial function independently of its blood pressure lowering action (Pelliccia, et al. 2010). Additionally, it has also been shown to play a role in lipid and glucose metabolism (Tuck 2005).

Cytokines, sensitive markers of tissue damage, are responsible for a negative inotropic effect in the failing human heart (Escobar, et al. 2004, Iqbal, et al. 2008) and in the pathophysiology of dilated cardiomyopathy (Tuck 2005). The increase in proinflammatory cytokines (IL-6) and oxidative stress markers (Thompson, et al. 2010) after EPI administration confirms that systemic inflammation/oxidative stress plays a central role in the cardiac damage induced by EPI. Indeed, significant correlations between cytokines/ROS levels and SR decline, observed in our previous study (Mantovani, et al. 2008) and confirmed in the placebo arm of the present trial, suggest that an increase in inflammatory/ROS markers may be analogous to early myocardial cell dysfunction shown by TDI.

A pathogenetic hypothesis based on oxidative stress has gained the widest acceptance in the study of acute EPI-induced CTX. Its molecular basis is attributable to the one-electron redox cycling of the quinone moiety, which generates ROS in excess of limited cardiomyocyte antioxidant defenses (Minotti, et al. 2004). This cellular pathway results in severe oxidative stress and disruption of the mitochondrial energetic machinery, ultimately leading to cardiomyocyte apoptosis or necrosis (Conklin 2005). Indeed, a relationship was found between cytokine release and ROS increase in patients with dilated cardiomyopathy (Kaur, et al. 2006).

The observation that telmisartan is able to prevent, at dosages over 200 mg/m^2^ EPI, such a number of potentially harmful effects induced by EPI, to which, however, its antineoplastic therapeutic efficacy is attributable, suggests that its administration may also compromise or weaken the antitumor efficacy of ANT. To date, however, this hypothesis is not supported by any data in the literature.

Regarding the use of telmisartan as a cardioprotective drug in our present trial, it is to be noted that recently (July 2010), and long after the beginning of our study (end of 2008) and concomitantly with the publication of the first results (September 2010), a meta-analysis was published by Sipahi et al. (Sipahi, et al. 2010). The authors concluded that when the analysis was limited to telmisartan, the excess in new cancer (lung cancer) occurrence was of borderline significance (p = 0.05) and that no statistically significant difference in cancer deaths was observed. Furthermore, the telmisartan dose used in the trials which were reviewed in the meta-analysis was 80 mg/day, i.e., double the dose used in our trial. For these reasons we believe that the findings of Sipahi et al. have no bearing on our study.

The potential shortcoming of the present study is the limited number of patients included: therefore, a confirmatory phase III randomized multicenter and possibly multinational trial is warranted.

In conclusion, the present study strengthens the findings of our earlier research (Cadeddu, et al. 2010) which aimed to assess the cardioprotective effect of telmisartan only during the period of EPI administration. It highlights that the protection obtained with the AT1R blockade has a long-lasting effect, probably by ensuring a permanent (at least up to 18-month FU) defense against chronic or late-onset types of ANT-induced CTX. This finding is extremely important, since ANT-induced CTX persists for years with no clinical symptoms, whereas upon the development of overt heart failure, the prognosis becomes extremely poor, possibly even worse than that of ischemic or idiopathic dilated cardiomyopathy (Cardinale, et al. 2008, Cardinale, et al. 2013 Colombo and Cardinale 2013).

The future potential development of the present study is thus to continuously monitor these two group of patients to evaluate whether the global cardiac function in the long term, at least for 3 years, is comparable between the two groups or whether the group protected with telmisartan has a better global cardiac function and thus a better clinical outcome. For this purpose the study is still in progress.
